# Improving the image quality of short-time bone SPECT using cadmium-zinc-telluride detectors with SwiftScan

**DOI:** 10.22038/aojnmb.2024.76919.1543

**Published:** 2025

**Authors:** Kazuto Funakoshi, Tomohiko Yamane, Eito Kozawa, Ichiro Matsunari

**Affiliations:** 1 Department of Radiation Oncology, Saitama Medical University Hospital, Saitama, Japan; 2 Department of Molecular Imaging Research, Kobe City Medical Center General Hospital, Hyogo, Japan; 3 Department of Nuclear Medicine, International Medical Center, Saitama Medical University, Saitama, Japan; 4 Department of Radiology, Saitama Medical University Hospital, Saitama, Japan; 5 Department of Nuclear Medicine, Saitama Medical University Hospital, Saitama, Japan

**Keywords:** Short-time bone SPECT/CT, CZT semiconductor camera, SwiftScan

## Abstract

**Objective(s):**

This study aimed to evaluate the quality and associated quantitative values of bone single-photon emission computed tomography (SPECT) with and without SwiftScan using a semiconductor camera equipped with a cadmium-zinc-telluride detector.

**Methods:**

Ten patients with bone metastases from prostate cancer who underwent list-mode SPECT/computed tomography using a whole-body semiconductor camera participated in this study. A total of 130 metastatic lesions from 10 patients were analyzed. Standard SPECT images were obtained approximately 3 h later, and the images were constructed with and without SwiftScan.

**Results:**

The visual assessment of 3-dimensional maximum intensity projection images showed that when an image quality score of 4 (good) or better was considered clinically acceptable, it was maintained at 4 or better in the 75% and 50% scans with SwiftScan, whereas only the 75% scan was considered acceptable without SwiftScan. The intraclass correlation coefficient was 0.952 at 5% for the standard time without SwiftScan and 0.990 with SwiftScan. The maximum standardized uptake value (SUV_max_) changes were 0 to 9.5 (median 1.1) at 75%, 0.1 to 11.5 (1.65) at 50%, 0 to 15.7 (2.1) at 25%, 0.1 to 33.2 (4.2) at 10%, 0.2 to 8.9 (5.65) at 5% without SwiftScan. On the contrary, the SUV_max_ changes in absolute value were 0 to 5.4 (median 0.8) at 75%, 0 to 6.5 (1.4) at 50%, 0 to 19.1 (1.7) at 25%, 0 to 24.2 (2.8) at 10%, 0 to 29.9 (2.6) at 5% with SwiftScan. The contrast-to-noise ratios (CNR) were 95.3 at 75%, 88.3 at 50%, 69.2 at 25%, 45.7 at 10%, and 31.6 at 5% without SwiftScan, and 96.9, 91.7, 78.0, 71.6, and 62.0, respectively, using SwiftScan.

**Conclusion:**

With the use of SwiftScan, a 50% reduction in acquisition time was considered acceptable for image quality with reproducible quantitative indices such as SUV_max_ and CNR.

## Introduction

 Patients with localized prostate cancer have a 5-year survival rate of >99 %. The survival rate decreases to 50% with the development of distant metastases. Prostate cancer progresses relatively slowly, with the bone being the most common site of distant metastasis. In addition to morphological evaluation methods, such as simple radiography, computed tomography (CT), and magnetic resonance imaging (MRI), metabolic evaluation methods, including bone scintigraphy and positron emission tomography/ CT (PET/CT), using tracers such as sodium fluoride (NaF), fluorodeoxyglucose (FDG), or prostate-specific membrane antigen (PSMA), have been used. In particular, bone scintigraphy is widely used because of its simplicity in identifying the presence and extent of metastasis and in determining treatment efficacy (1, 2). PET/CT is also considered useful but is less widely used than bone scintigraphy (3). Bone metastases from prostate cancer tend to show osteogenic changes and are easy to detect by bone scintigraphy owing to their increased accumulation (4, 5).

 Recently, with the widespread use of semiconductor gamma cameras, short-time bone single-photon emission computed tomography (SPECT) evaluation of bone metastases has been reported (6, 7). Although SPECT or SPECT/CT imaging improves diagnostic performance, the addition of SPECT images increases the total scan time. Patients with bone metastases typically undergo frequent bone scintigraphy; therefore, it is important to minimize radiation exposure and perform examinations in a short time. 

 Furthermore, long scanning times are often associated with discomfort, particularly for sick patients. Compared to conventional scintillation cameras, semiconductor cameras with cadmium-zinc-telluride (CZT) detectors have high energy and spatial resolution; therefore, they reduce the amount of contrast agent injected and shorten the test time (1) (8, 9). 

 Clinical evaluation of short-term bone SPECT/CT acquisition with whole-body CZT detectors has been reported (10). Furthermore, the gamma camera of GE Healthcare is equipped with a new feature called SwiftScan that allows data to be collected while the detector is moving. Simulations of human phantoms using CZT cameras have been reported (11, 12), but no clinical applications have yet been reported. 

 The use of SwiftScan may further reduce collection time; however, the aforementioned study (10) did not examine this function or its quantitative effects. Therefore, this study aimed to evaluate the quality and associated quantitative value of the SwiftScan in short-time bone SPECT using a CZT camera.

## Methods

***Patients***

 Ten patients with bone metastases from prostate cancer who underwent list-mode SPECT/CT imaging with a whole-body semiconductor camera at our hospital between December 2019 and February 2022 were included. The 130 lesions of 10 patients from the 150 lesions of 13 patients in a previous study (Yamane et al. Sci Rep, 11(1): 24320) were evaluated. The number of patients analyzed was 76.9%, and 86.6% of the lesions were analyzed. Because a long acquisition time is usually mandatory in standard SPECT imaging protocols, we evaluated the data of 10 patients for whom two or more table positions were necessary for SPECT acquisition, which is inconvenient for sick patients.

 This retrospective study was approved by the Ethical Review Board of Saitama Medical University Hospital (numbered 2021-138 dated March 4, 2022), and the requirement for written informed consent was waived.

***Bone SPECT/CT imaging***

^99m^Tc-methylene diphosphonate (PDR Radio-pharma, Inc. Tokyo, Japan) was injected intravenously and a standard target dose of 740 MBq was administered. The actual injected dose was calculated by measuring the dose in the syringe before and after the injection. After approximately 3 h, the patients were asked to void and images were acquired using a whole-body semiconductor camera (GE Healthcare, Chicago, IL, USA. Discovery NM/CT 670 CZT scanner). Standard-scan SPECT/CT images were acquired using a parallel flat board collimator with a non-circular orbit, wide energy range, and high resolution. Images were collected using a step-and-shoot method that rotated 360° in a noncircular orbit, and 60 locations were collected at 6° each during rotation. Unlike the conventional frame mode, in which the γ-ray counts are accumulated on predetermined position coordinates in memory, the collection was performed in list mode, which records the time, position, and energy of each incident γ-ray, in addition to storing the position information directly from the detector. SwiftScan combines a low-energy high-resolution collimator with the tomography mode. In SPECT acquisition, in addition to the usual step-and-shoot acquisition every 6°, the collection of 3° immediately before and after the rotation was assigned to the collection every 6° before and after the rotation. The energy range was 140 keV ±7.5%. The collection time for each table was approximately 10 min. All SPECT images were reconstructed using the ordered subset expectation-maximization (OSEM) approach. The number of iterations was 4, the subset was 10, the matrix size was 128×128, and the voxel size was 4.42×4.42×4.42 mm. The ASiR reconstruction technique (GE Healthcare) was used to obtain CT images. The parameters were 120 keV, auto mA, noise index 35, 512×512 matrix, 1.375 pitch, and 0.5-second rotation. The first 5, 10, 25, 50, 75, and 100% of the images in the list-mode data were reconstructed on a workstation (GE Healthcare. Xeleris ver. 4.1) from 100% images. Three-dimensional maximum intensity projection (3D-MIP) images were generated to determine the overall image quality. Images were captured with and without the Swift Scan.

***Image quality assessment***

 Visual evaluation of the 3D-MIP images was performed by two observers using a 5-point scale with 5, 4, 3, 2, and 1 representing “excellent”, “good”, “fair”, “poor”, and “very poor”, respectively Disagreements between the two observers were resolved by consensus.

***Setting of the region of interest***

 The region of interest (ROI) was defined as the area of increased accumulation reflective of bone metastases on standard images. The maximum standardized uptake value (SUV_max_) was measured for each ROI. The reference area was set at the proximal femur, which is away from the metastatic lesion. Two experts in nuclear medicine defined the ROI. The ROI positioning and SUV_max_ calculations were performed using a workstation (Xeleris).

***Statistical analysis***

 The intraclass correlation coefficients (ICC) of SUV_max_ for each reconstructed percentage and normal examination time, with and without SwiftScan, were examined. In a previous study (10), the ICC value was > 0.8, indicating a near-perfect correlation. To evaluate these differences, a Bland–Altman plot was constructed for each patient and the percentage change in each SUV_max_ was calculated. The contrast-to-noise ratio (CNR) was calculated to evaluate image quality using the following formula:

## Results

**Figure 1:**
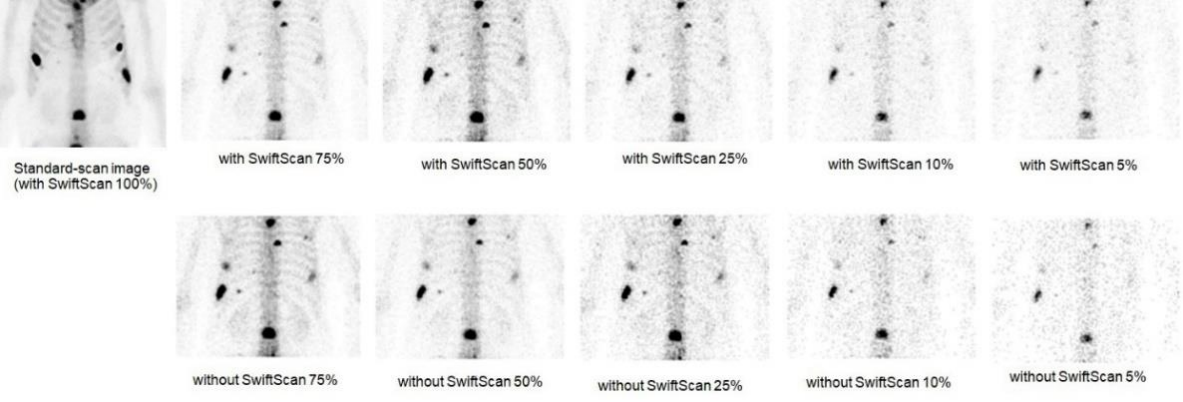
Standard-scan image and short-time imaging scan with and without SwiftScan

 A total of 130 metastatic lesions were analyzed in 10 patients. The doses injected ranged from 662.3 to 878.1 MBq, with a median of 732.8 MBq. The time from injection to the start of testing ranged from 165 to 220 min, with a median time of 200.5 min (Table 1). The obtained SPECT images are shown in Figure 1. Images captured with and without SwiftScan were composed for each period, with 100% of the images captured at the standard time using SwiftScan.

**Table 1:** Patient characteristics

**Patient no**	**Age (years)**	**Dose (MBq)**	**SPECT table position**	**Time between tracer injection**	**No. of measurement**
			**number of bed positions**	**SPECT start (min)**	**number of lesions**
1	82	878.1	3	206	21
2	76	739.6	2	194	11
3	69	662.3	2	207	11
4	65	868.9	2	200	21
5	83	687.0	3	204	8
6	72	673.0	2	197	7
7	74	699.0	2	198	10
8	76	764.9	2	220	11
9	74	726.0	2	201	18
10	79	765.1	2	165	12
Median	75	732.8	2	200.5	11

The images were obtained within a short acquisition time with and without SwiftScan, wherein normal imaging with SwiftScan was used as a reference point (100%)

**Figure 2:**
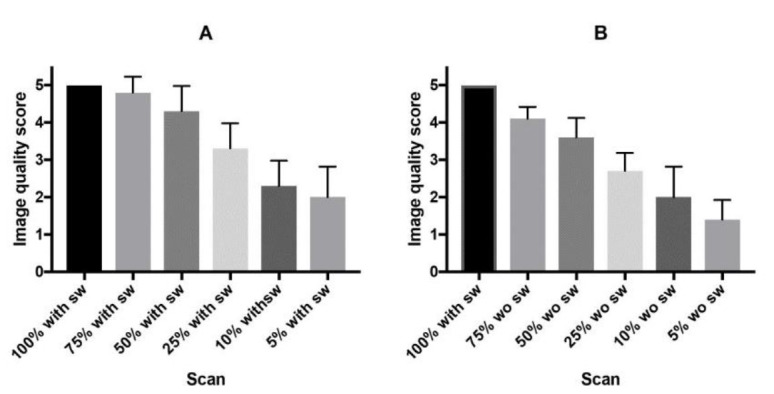
Image quality scores with (**A**) and without (**B**) SwiftScan

 The results of the image quality assessment of the 3D-MIP images are summarized in Figure 2. On visual assessment, the image quality of the 100% scan with SwiftScan was rated 5 in all 10 patients. The image quality score decreased as a function of imaging time, with and without SwiftScan. However, when an image quality score of 4 (good) or better was considered clinically acceptable, it was maintained at 4 or better in the scans at 75% and 50% duration images with SwiftScan, whereas only the scans at 75% duration images was considered acceptable without SwiftScan.

**Figures 3, 4:**
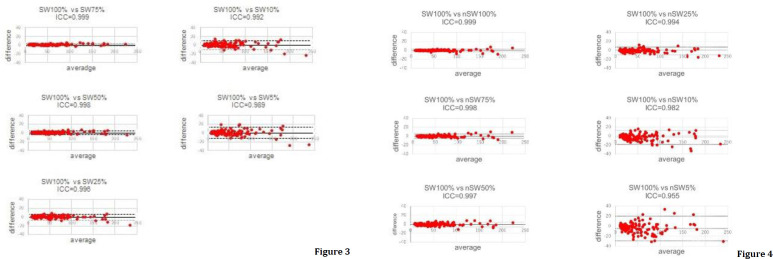
Bland–Altman plots for the images with (**Figure 3**) and without (**Figure 4**) SwiftScan

 The ICC value was as high as 0.952, even at 5% relative to the standard time, without SwiftScan, and remained even higher, at 0.990, with SwiftScan (Table 2). The Bland–Altman plots, with and without SwiftScan, for each acquisition time versus the standard acquisition time are shown (Figures 3 and 4).

**Table 2:** Intraclass correlation coefficient (ICC)

**Against standard acquisition time**	**With SwiftScan**	**Without SwiftScan**
**(With SwiftScan as 100%)**		
100%	-	0.999
75%	0.999	0.998
50%	0.999	0.997
25%	0.997	0.994
10%	0.993	0.982
5%	0.990	0.952

The difference between the measured values of the short-time acquisition images with and without SwiftScan and the respective ICC values, with the reference image with SwiftScan, was set to 100%. The solid lines represent the bias lines and the dashed lines represent the upper and lower confidence intervals

Horizontal dashed lines are 95% confidence intervals

ICC, Intraclass correlation coefficient

**Figure 5:**
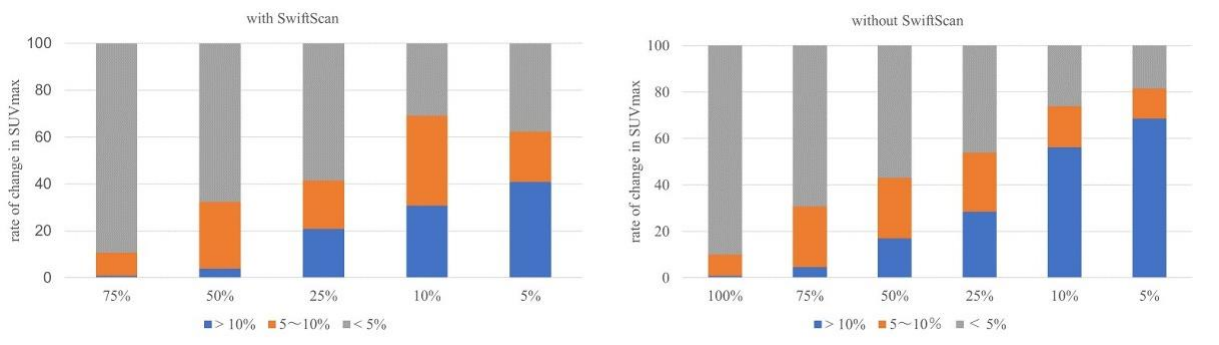
Rate of change in SUV_max_

The percentage of SUV_max_ changes >10% without SwiftScan was 0.7% for full-duration images in the list-mode data, 4.6% at 75%, 16.9% at 50%, 28.5% at 25%, 56.2% at 10%, and 68.4% at 5%-duration images. In contrast (Figure 5), SwiftScan suppressed the rates to 0.75% at 75%, 3.85% at 50%, 20.8% at 25%, 30.8% at 10%, and 40.8% at 5%-duration images.

**Figure 6:**
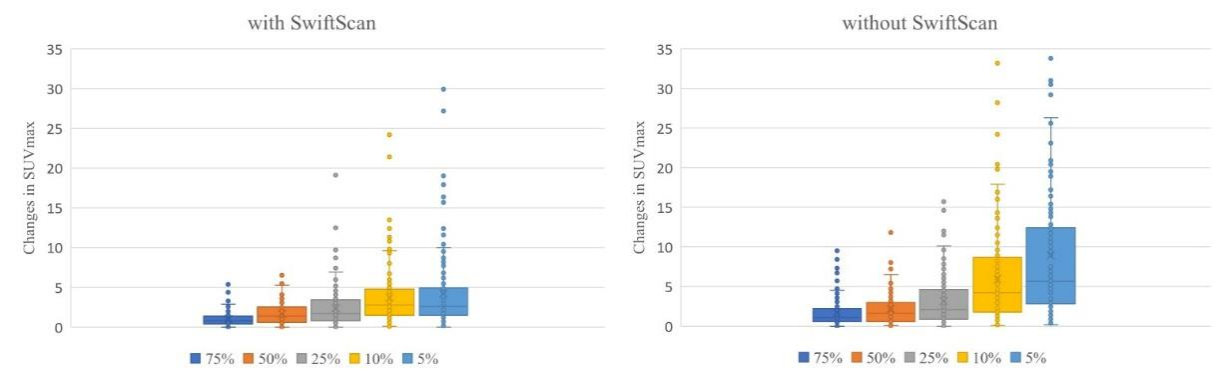
Actual changes in SUV_max_

**Figure 7:**
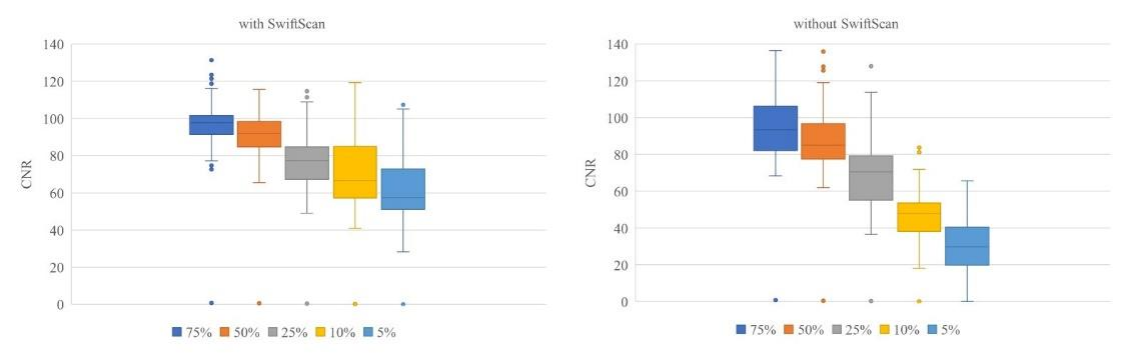
A box-and-whisker plots of CNR

 The percentage change based on full-duration images using SwiftScan is shown in Figure 5. As shown in Figure 6, the SUV_max_ changes from 0 to 9.5 (median 1.1) at 75%, 0.1 to 11.5 (1.65) at 50%, 0 to 15.7 (2.1) at 25%, 0.1 to 33.2 (4.2) at 10%, 0.2 to 8.9 (5.65) at 5%-duration images without SwiftScan. In contrast, the SUV_max_ changes in absolute values were from 0 to 5.4 (median 0.8) at 75%, 0 to 6.5 (1.4) at 50%, 0 to 19.1 (1.7) at 25%, 0 to 24.2 (2.8) at 10%, 0 to 29.9 (2.6) at 5%-duration images with SwiftScan. CNR was 95.3 in images captured at 75% of the standard duration, 88.3 at 50%, 69.2 at 25%, 45.7 at 10%, 31.6 at 5%-duration images, and the SwiftScan maintained high values at 96.9, 91.7, 78.0, 71.6, and 62.0 respectively (Table 3). Figure 7 shows the CNR. 

 CNR did not differ significantly with or without SwiftScan if the acquisition time was sufficiently long; however, for shorter acquisition times, CNR was better with SwiftScan than without SwiftScan.

The changes in SUV_max_ during short-time acquisition with and without SwiftScan are shown. Changes >10% are shown in light blue, changes in the range of 5–10% are shown in orange, and changes < 5% are shown in gray, using the standard test with SwiftScan as 100%

SUV_max_, maximum standardized uptake value

The upper and lower whiskers indicate the 10th and 90^th^ percentiles of the data, respectively

SUV_max_, maximum standardized uptake value

**Table 3:** Contrast to Noise Ratio

**Against standard acquisition time**	**With SwiftScan**	**Without SwiftScan**
**(With SwiftScan as 100%)**		
100%	-	97.9
75%	96.9	95.3
50%	91.7	88.3
25%	78.0	69.2
10%	71.6	45.2
5%	62.0	31.6

## Discussion

 The main results of the study are as follows:

1. Visual analysis showed that when an image quality score of 4 (good) or better was considered clinically acceptable, it was maintained at 4 or better in the 75% and 50%-duration images with SwiftScan, whereas only the 75%-duration images was considered acceptable without SwiftScan.

2. ICC values show no difference with or without SwiftScan for long-duration examinations, but higher values are obtained with SwiftScan for shorter acquisition times.

3. Although the rate of change in SUV_max _increased with a short acquisition time, with or without SwiftScan, the use of SwiftScan reduced the high rate of change.

4. CNR did not differ significantly with or without SwiftScan if the acquisition time was sufficiently long; however, for shorter acquisition times, CNR was better with SwiftScan than without SwiftScan.

 The results of the visual assessment of image quality indicate that when the image quality was "good" or better was considered acceptable, the SwiftScan allowed a 50% reduction in acquisition time whereas the acceptable reduction was 25% without SwiftScan. Thus, using SwiftScan was beneficial in reducing the acquisition time in bone SPECT.

 The ICC is an indicator used to evaluate reproducibility. These values were high, regardless of the presence or absence of SwiftScan. Considering that values of 0.75 or higher indicate good reproducibility, the values obtained in our study can be considered to have high reproducibility. SUV_max_ is widely used and reproducible. It can be measured independently of the ROI size (2) and has been used to predict bone metastasis (4). Whereas the CNR showed an increase in background noise with a reduction in acquisition time with and without the SwiftScan, the rate decreased by 10% without the SwiftScan and remained relatively constant up to 5% with the SwiftScan. Short-term images obtained without SwiftScan had inferior image quality, which increased the possibility of false positives. Notably, SwiftScan provides sufficiently good image quality, even with a short scanning time. A short acquisition time is desirable for patients with bone metastases, as it allows the examiner to perform an increased number of examinations. In addition, the number of injected radiopharma-ceuticals is reduced, and for patients who are likely to be tested multiple times, this reduces radiation exposure. Although it is possible to collect sufficient data approximately 25% of the time (6), the initial diagnosis encompasses approximately 75% of the total acquisition time, and a short acquisition time may be desirable for follow-up. Normally, one step every 6° required 20 s to complete. This included a collection time of 17 s and a move time of 3 s. Because the collection was performed at 360° with two detectors, one detector required approximately 600 s for 30 steps. The collection time was 510 s, and the movement time was 90 s. As the collection time at 100% was 510 s, SwiftScan should reduce the collection time by this amount. At 75%, the collection time was 382 s and the travel time was 90 s for a total of 472 s. Similarly, at 50%, 255 s + 90 s for 345 s. At 25%, 127 s + 90 s for a total of 217 s. At 10%, 51 s + 141 s for 191 s. At 5%, the time was shortened to 25 s + 90 s for a total of 117 s. In this study, we found that the addition of 90 s to each scan was substantial.

 The ICC values with or without SwiftScan provided sufficient evaluation results with a short acquisition time. However, with shorter collection times, SwiftScan increased reproducibility.

 SUV_max_ is associated with a higher rate of change as the acquisition time shortens, but the rate of change decreases when SwiftScan is applied; therefore, SwiftScan should be used for short acquisition time.

 The CNR indicates that the noise tends to increase as the acquisition time decreases. Therefore, the shorter the acquisition time, the better the images obtained using SwiftScan.

 Currently, there are no firm indicators on SwiftScan, but it can be considered as one of them.

 A limitation of this study is that owing to the paucity of cases, a histological examination of patients with prostate cancer bone metastases was not performed. Bone metastases from prostate cancer are predominantly osteogenic lesions, but there is a large proportion of osteolytic lesions as well, and the results may differ for mixed lesions (13). Further research is required to assess whether this approach can be applied to other cancer types.

## Conclusion

 With the use of SwiftScan, a 50% reduction in acquisition time was considered acceptable for image quality with reproducible quantitative indices such as SUV_max_ and CNR.
